# The Korean Mistletoe (*Viscum album coloratum*) Extract Has an Antiobesity Effect and Protects against Hepatic Steatosis in Mice with High-Fat Diet-Induced Obesity

**DOI:** 10.1155/2013/168207

**Published:** 2013-07-11

**Authors:** Hoe-Yune Jung, Yu-Hee Kim, In-Bo Kim, Ju Seong Jeong, Jung-Han Lee, Myoung-Sool Do, Seung-Pil Jung, Kwang-Soo Kim, Kyong-Tai Kim, Jong-Bae Kim

**Affiliations:** ^1^School of Life and Food Sciences, Handong Global University, Pohang, Gyeongbuk 791-708, Republic of Korea; ^2^Research and Development Team, Pohang Center for Evaluation of Biomaterials, Pohang, Gyeongbuk 790-834, Republic of Korea; ^3^Division of Integrative Biosciences and Biotechnology (IBB), POSTECH (WCU), Pohang, Gyeongbuk 790-784, Republic of Korea; ^4^Department of Family Medicine, Yeungnam University College of Medicine, Daegu 705-717, Republic of Korea; ^5^Molecular Neurobiology Laboratory, McLean Hospital, Harvard Medical School, Belmont, MA 02478, USA; ^6^Department of Life Science, Division of Molecular and Life Science, POSTECH, Pohang, Gyeongbuk 790-784, Republic of Korea

## Abstract

This study investigates the inhibitory effects of Korean mistletoe extract (KME) on adipogenic factors in 3T3-L1 cells and obesity and nonalcoholic fatty liver disease (NAFLD) in mice fed a high-fat diet. Male C57Bl/6 mice fed a high-fat diet were treated with KME (3 g/kg/day) for 15 weeks for the antiobesity and NAFLD experiments. Body weight and daily food intake were measured regularly during the experimental period. The epididymal pad was measured and liver histology was observed. The effects of KME on thermogenesis and endurance capacity were measured. The effects of KME on adipogenic factors were examined in 3T3-L1 cells. Body and epididymal fat pad weights were reduced in KME-treated mice, and histological examination showed an amelioration of fatty liver in KME-treated mice, without an effect on food consumption. KME potently induces mitochondrial activity by activating thermogenesis and improving endurance capacity. KME also inhibited adipogenic factors *in vitro*. These results demonstrate the inhibitory effects of KME on obesity and NAFLD in mice fed a high-fat diet. The effects appear to be mediated through an enhanced mitochondrial activity. Therefore, KME may be an effective therapeutic candidate for treating obesity and fatty liver caused by a high-fat diet.

## 1. Introduction

Obesity is the most common metabolic disorder and results from the combined effects of excess energy intake and reduced energy expenditure [1]. It is one of the fastest growing major disorders throughout the developed nations [2]. It is associated with a variety of clinical disorders in developed nations [2]. Obesity is associated with a variety of clinical disorders, including hypertension, insulin resistance, glucose intolerance, and dyslipidemia. It is well known that an oversupply of fat is associated with the development of obesity in mice [3]. Long-term feeding with a high-fat diet can induce obesity with hyperlipidemia, insulin resistance, hyperphagia, and hypergluconemia [4, 5].

Nonalcoholic fatty liver disease (NAFLD) is present in up to one-third of the general population and affects all ages and ethnic groups. NAFLD is the second leading cause of death in the general population [6, 7]. At present, there is no pharmacological agent known to reverse NAFLD and effective medical interventions have focused on the modification of risk factors, such as weight reduction and diet [8].

Mistletoe is a hemiparasitic plant growing all over the world on various deciduous trees, such as oak. It has been used as a traditional herbal medicine, especially in Europe [9]. Korean mistletoe (KM, *Viscum album cololatum*) extract (KME) has been shown to have anticancer [10], antioxidant [11], antidiabetes [12] and antidementia [13] effects, and it enhances immune system function [14]. Recently, we reported that KME significantly increases mitochondrial function [15].

Mitochondria are known as “cellular power plants” because they generate most of the cellular supply of adenosine triphosphate (ATP) which is used as a source of chemical energy. Studies have implicated mitochondria in several human diseases, including metabolic diseases [16], cardiac dysfunction [17], mental disorders [18], and the aging process [19]. Decreased mitochondrial respiration rates [20] and reduced expression of genes involved in mitochondrial oxidative capacity [21] have been reported in diet-induced obese rats. Because KME improves mitochondrial function, we asked whether it has beneficial effects on obesity.

It is well known that adipocyte differentiation and the extent of subsequent fat accumulation are closely related to the occurrence and advancement of various diseases, such as coronary artery disease and obesity [1, 22, 23]. 3T3-L1 cells have served as a useful *in vitro* model for adipocyte differentiation and function [24]. The differentiation of preadipocytes into adipocytes requires a variety of effectors that activate a cascade of transcription factors, such as CCAAT/enhancer-binding protein-*α* (C/EBP-*α*), peroxisome proliferator-activated receptor*γ* (PPAR-*γ*), and sterol regulatory element binding element protein-1c (SREBP-1c). This cascade begins with the CCAAT enhancer-binding protein- (C/EBP-) *β* and *δ*, which induces the expression of C/EBP-*α* and PPAR-*γ* [25–27]. These transcription factors coordinate the expression of genes involved in creating and maintaining the adipocyte phenotype, including genes for adipocyte fatty acid-binding protein, glucose transporter 4, lipoprotein lipase (LPL) and leptin [28, 29]. SREBP-1c is strongly associated with SREBP cleavage-activating protein (SCAP). Activated SREBP-1c accelerates adipogenesis through the overexpression of adipogenic enzymes, such as fatty acid synthase(FAS), acyl-CoA synthase (ACC), and acyl-CoA carboxylase (ACS). LPL is the major enzyme that hydrolyzes triglyceride (TG) molecules of chylomicrons and VLDL particles. The released free fatty acids are either oxidized to generate ATP in muscle or stored in adipose tissue [30].

We measured the inhibitory effect of KME on adipogenic factors in 3T3-L1 cells and on the development of obesity and NAFLD in mice fed a high-fat diet. Our data show that KME suppresses adipocyte differentiation through down-regulation of adipogenic factors and could ameliorate obesity and NAFLD in mice fed a high-fat diet. Our results indicate the great potential of KME as a potential metabolic regulator of adipocyte differentiation and a potential therapeutic agent for preventing or treating obesity and NAFLD.

## 2. Materials and Methods

### 2.1. Preparation of KME

Mistletoe growing on oak was harvested from Gangwon-do, Republic of Korea, in February. The mistletoe was 1 or 2 years old, and the leaves, stems, and fruits were cut into 2 joints from the end of a branch, washed with distilled water (DW), and dried. The vacuum-packed mistletoe was stored at −80°C until extracted. Then mistletoe leaves, fruits, and stems were freeze-dried, crushed and ground in approximately 10 volumes of DW for 30 seconds. After being washed, they were ground in a mixer for 2 minutes and stirred for 16 hours at 4°C. To obtain fine mistletoe extract, the homogenized mistletoe was centrifuged at 8,000 rpm for 30 minutes at 4°C, and the resulting supernatant was successively filtered through different pore sizes (0.9 and 0.45 *μ*m). The mistletoe extract was freeze-dried and resuspended in DW at an appropriate dilution factor.

### 2.2. Animals and Diets

Lean, male C57BL/6 mice (7 weeks old) were purchased from Central Lab Animal Inc. (Seoul, Republic of Korea). All mice were housed for 1 week under a 12/12-hour light/dark cycle in a temperature—(22 ± 1°C) and humidity—(55 ± 5%) controlled room and fed standard laboratory chow and water ad libitum. KME was mixed with either powdered chow (M07, Feedlab, Republic of Korea) or high-fat (D12327, Research Diets, Inc., New Brunswick, NJ, USA) feed at a concentration of 4 g/kg of food to provide a 3000 mg/kg/day (mpk) dose. The powdered chow was then made into pellets. Control groups received pellets without KME. These dietary amounts represent the maximum amount of chow that these animals were able to consume during a 24-hour period. Seven C57BL/6 mice on the above *ad libitum *diets had their daily average caloric intake and weekly body weight measurement during the course of the study. At the conclusion of the *in vivo* experiment, the mice were sacrificed and the epididymal pads were collected and weighed.

### 2.3. Cold Test

The adaptive thermogenic response was measured in a cold test, during which the animals were individually housed at 4°C for 6 hours [31].

### 2.4. Liver Histology

Liver tissues were isolated immediately after sacrifice. For hematoxylin and eosin (HE) staining, tissues were fixed in 10% formalin and processed and embedded in paraffin prior to sectioning (10 *μ*m.) and staining. Liver biopsies for electron microscopy were cut into 1 mm pieces, fixed immediately after collection in Karnovsky fixative (glutaraldehyde in cacodylate buffer), and kept at 4°C. The second step involved postfixation with 1% osmium tetraoxide in 0.1 M cacodylate buffer for 1 hour at 4°C. Tissues were then dehydrated through successive baths of graded alcohol, followed by a propylene oxide bath and a treatment with propylene oxide and resin mix before being embedded in a pure epoxy resin (araldite, Epon 812) that solidified after 48 h at 60°C. Semithin sections were cut at 2 *μ*m, stained with toluidine blue, and then analyzed by light microscopy. Ultrathin sections were cut at 70 nm and examined with an electron microscope (BX 50, Olympus, Japan). Electron micrographs (400x magnification) were digitized and analyzed using ACD SEE, version 4.0. The liver of 1 mouse from each group (control, high-fat diet (HFD), HFD + KME 3000 mg/kg) was measured. In brief, the following criteria were used for scoring hepatic steatosis: grade 0, grade 1, hepatocytes occupying <33% of the hepatic parenchyma; grade 2, fatty hepatocytes occupying 33–66% of the hepatic parenchyma; and grade 3, fatty hepatocytes occupying >66% of the hepatic parenchyma.

### 2.5. Endurance Test

**Figure 1:**
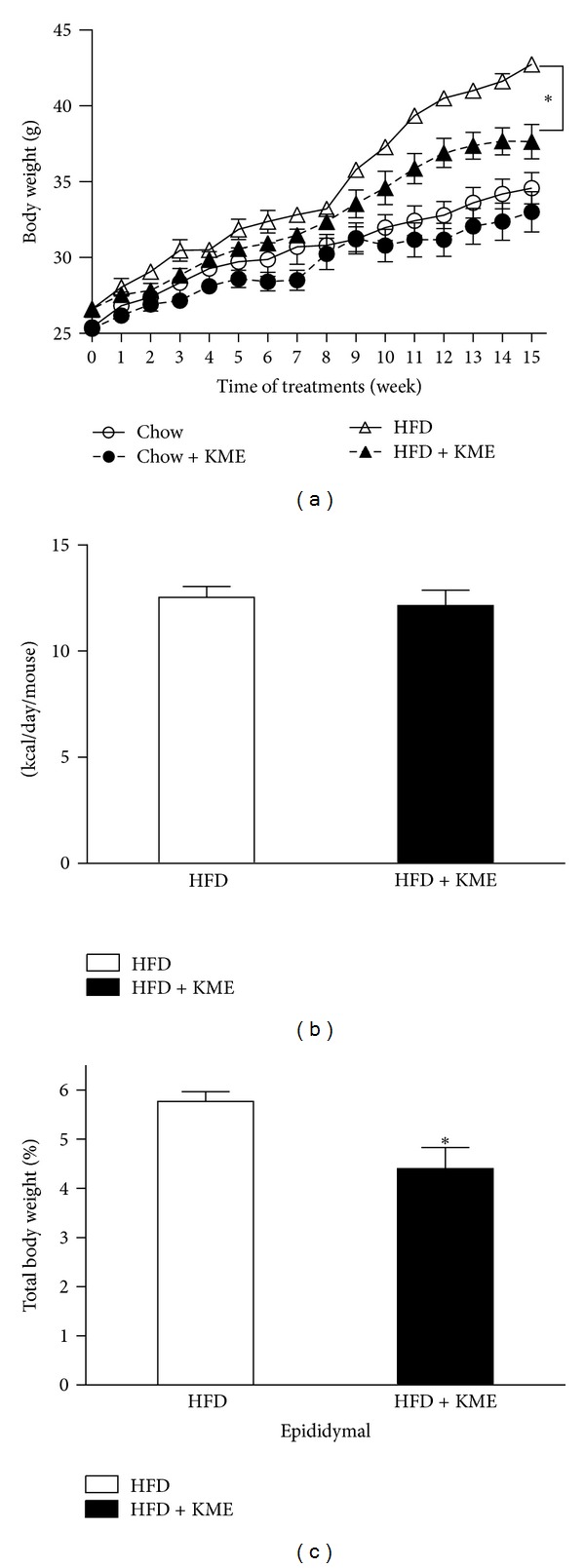
Effect of KME on body weight and food intake in mice fed a high-fat diet for 15 weeks. (a) Changes in body weight gain at each treatment period are shown. (○) C: chow diet; (●) C + KME: chow diet plus KME 3000 mg/kg; (△) HFD: high-fat diet; (▲) HFD + KME: high-fat diet plus KME 3000 mg/kg. (b) Average food intake expressed as kcal/mouse/day. (c) Epididymal fat weight expressed as percentage of total body weight. Values represent mean ± standard error of mean (SEM; *P < 0.05, *n* = 7 per group).

Mice were subjected to an endurance test using a variable-speed belt treadmill enclosed in a plexiglass chamber with a stimulus device consisting of a shock grid attached to the rear of the belt (Columbus Instruments Oxymax System, Columbus, OH, USA). The shock grid was set to deliver 0.2 mA, which caused an uncomfortable shock but did not physically harm or injure the animals. Animals were acclimatized to the test using a habituation protocol. Mice were run at 16.2 meter/minutes for 10 minutes with a 5° incline. For high-fat- (HF-) fed animals, the experiment was initiated at 10.8 meter/minutes at a 0° incline. The speed was gradually increased from 10.8 to 24.6 meter/minutes and then maintained until exhaustion [15]. Exhaustion was determined to have been reached if the mice were unable to run for 10 seconds [32], at which point the electric shock was discontinued.  Figure 1 shows the running test protocol.

### 2.6. Cell Culture and Induction of Adipocyte Differentiation

3T3-L1 preadipocytes purchased from ATCC (American Type Culture Collection, Manassas, VA, USA) were cultured in Dulbecco's-modified Eagle's medium (DMEM, Hyclone, Logan, UT, USA) containing 10% bovine calf serum (BCS, Hyclone, Logan, Utah, USA) at 37°C in a humidified atmosphere of 5% CO_2_. After 3 or 4 days, the cells had reached 90% confluence and were collected with 0.05% trypsin/0.53 mM EDTA treatment. After centrifugation (1300 rpm, 5 minutes), the cells were plated in 6-well plates at a concentration of 3 × 10^4^ cell/well. One day after confluence (designated “day 0”), cell differentiation was induced with a mixture of methylisobutylxanthine (0.5 mM), dexamethasone (0.25 mM), and insulin (5 mg/mL) in DMEM containing 10% fetal bovine serum (FBS). On day 2 and thereafter, DMEM supplemented with 10% FBS and 5 mg/mL insulin was replaced every 2 days. 3T3-L1 adipocytes, 7 to 8 days after differentiation, were treated with KME 6 *μ*g/*μ*L or phosphate-buffered saline (PBS) for 24 hours.

### 2.7. RNA Preparation and Real-Time RT-PCR

Total RNA was isolated from 3T3-L1 adipocytes using TRIzol reagent (Invitrogen, Carlsbad, CA, USA). Cells were homogenized by 4-5 passages through a 23-gauge needle. After a 5-minute reaction, samples were mixed with chloroform by vortex machine for 15 seconds. Samples were then centrifuged at 12,000 rpm for 10 minutes at 4°C. The colorless supernatant was transferred into new tubes. Total RNA was precipitated by mixing with isopropyl alcohol and centrifuging at 12,000 rpm. The resulting RNA pellets were washed with 75% ethanol and dissolved in RNase-free water. RNA was quantified by spectrophotometry at A_260_ and then stored at −80°C. Total extracted RNA (1 *μ*g) mixed with annealing oligo dT primer and RNase-free water was incubated in a thermocycler for denaturing RNA. Reactant was mixed with 5X first strand buffer, 20 mM DTT, 10 mM dNTP mix, RNase-free water, and Superscript II reverse transcriptase. The mixtures were incubated in a thermocycler (42°C for 1 hour and 72°C for 7 minutes) to generate cDNA. Real-time RT-PCR analysis was performed with an AB 7500 real-time PCR system (Applied Biosystems, Foster City, CA, USA). Samples containing 2X SYBR Green PCR Master Mix (Applied Biosystems, Foster City, CA, USA), 0.5 *μ*mol each of appropriate primers, and cDNA were incubated in the AB 7500 real-time PCR system for an initial denaturation at 94°C for 10 minutes, followed by 40 PCR cycles. Each cycle was run at 95°C for 15 seconds and at 60°C for 1 minute. The oligonucleotide primers for the experiment are listed in Table 1. *β*-actin was used as an internal housekeeping control. To confirm amplification of specific transcripts, melting curve profiles were produced at the end of each PCR cycle by cooling the sample to 65°C for 15 seconds and heating slowly to 95°C, with continuous fluorescence measurement.

### 2.8. Statistical Analysis

Data (means ± SE) were analyzed using GraphPad Prism (version 5.04, GraphPad Software, USA). Unpaired two-tailed Student's *t*-tests were used to evaluate differences between means, as indicated. *P* values < 0.05 were considered statistically significant.

## 3. Results 

### 3.1. KME-Induced Body Weight, Dietary Intake, and Adiposity Changes in HFD-Fed Mice

During the 15-week experiment, body weight and food intake were measured weekly. As shown in Figure 1, the KME-treated group had a slight decrease in body weight compared to the control group. However, this effect was significantly pronounced in animals that were fed an HF diet with KME. The difference between the KME-treated and control animals became significant at week 9. At the end of the experiment, the body weight of mice fed KME was 20% lower (*P* < 0.05) than that of the control group, whereas HF diet plus KME mice weighed almost the same as the mice that ate normal chow (Figure 1(a)). These effects of KME on body weight were not due to decreased food intake, as the amount of kcal consumed per mouse over a 24-hour period remained unchanged (Figure 1(b)). The data indicate that KME might have antiobesity effects *in vivo*, without affecting food intake.

To test whether body weight loss was caused by decreased adiposity, animals were sacrificed and epididymal white adipose tissue was dissected and weighed. This analysis revealed that epididymal white adipose tissue was significantly reduced in the HF diet plus KME group (Figure 1(c)).

### 3.2. Effect of KME on Cold Exposure in HFD-Fed Mice

**Figure 2:**
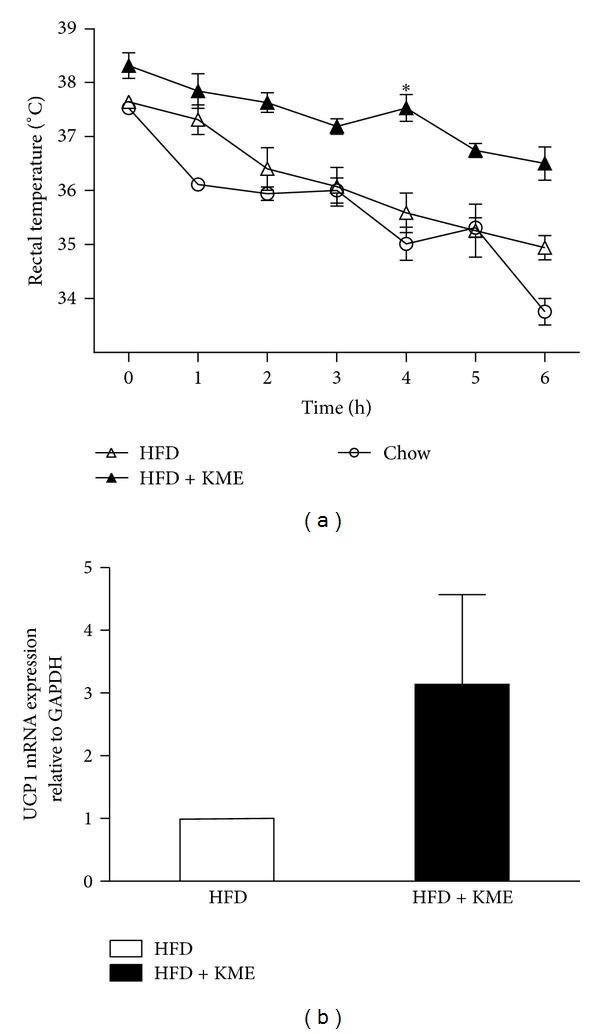
effect of KME on body temperature during the cold test (4°C for 6 h) and on UCP1 mRNA expression in BAT. (a) Hourly changes in body temperature are shown. (△) HFD: high-fat diet; (▲) HFD + KME: high-fat diet plus KME 3000 mg/kg (*P < 0.05, *n* = 7 per group). (b) The relative UCP1 mRNA expression level in BAT of animals treated with HFD or HFD + KME (high-fat diet plus KME 3000 mg/kg) as quantified by real-time PCR. Values represent mean ± SEM (*P < 0.05, *n* = 3).

We performed a cold test to assess the effect of KME on adaptive thermogenesis capacity. KME-treated animals maintained higher body temperatures than nontreated animals (Figure 2(a)), suggesting that it improved this capacity. To test whether thermogenesis is accompanied by changes in the expression of genes involved in thermogenesis, total RNA was prepared from BAT. We examined mRNA levels of UCP1, the major contributor to heat production, by Real-time RT-PCR.  Figure 2(b) shows that the mRNA level of UCP1 was remarkably increased in KME-treated mice.

### 3.3. Effect of KME on Endurance Capacity of HFD-Fed Mice

**Figure 3:**
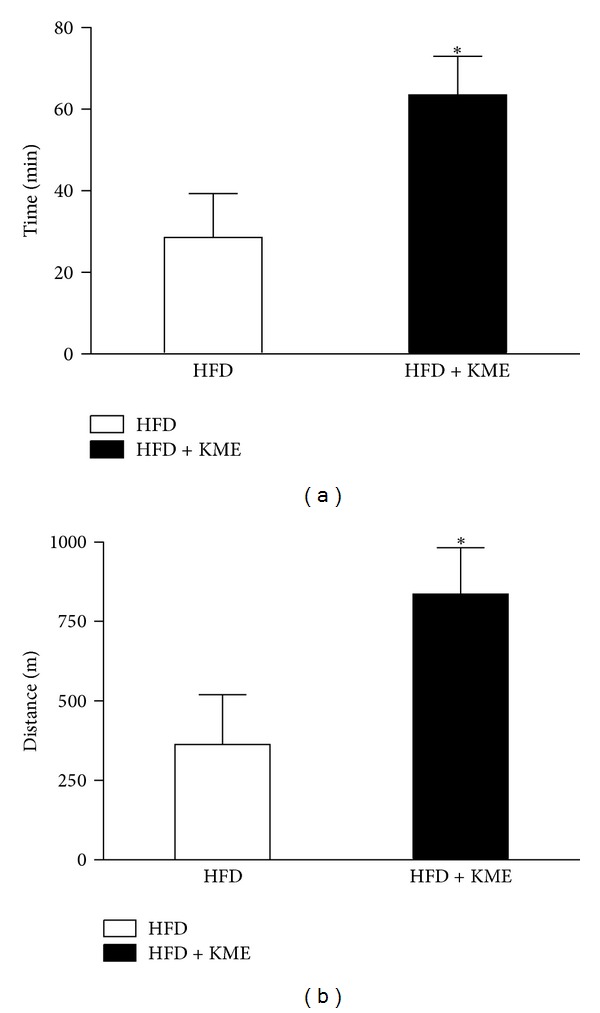
Endurance capacity was enhanced in KME-treated mice over 15 weeks. Average times (a) and distance (b) run until exhaustion are presented for animals treated with HF or HF + KME at 3000 mpk. Values represent the mean ± SEM (*P < 0.05, *n* = 7 per group).

Because KME reduced body weight (Figure 1(a)) and increased thermogenesis (Figure 3), we evaluated the effect of KME administration on a treadmill endurance test. The mice were trained to run at 16.2 meter/minutes for 10 minutes at a 5° incline the day before the running test, according to the procedure used for our previous report [15]. The experiment was initiated at 10 meter/minutes at a 0° incline with a gradual increase in speed. The mice were run until exhaustion, which was defined as remaining on the shock grid for longer than 10 consecutive seconds. Surprisingly, KME-treated mice ran twice as far as high-fat diet mice (Figure 3). 

### 3.4. Effect of KME on Hepatic Histology of HFD-Fed C57BL/6

**Figure 4:**
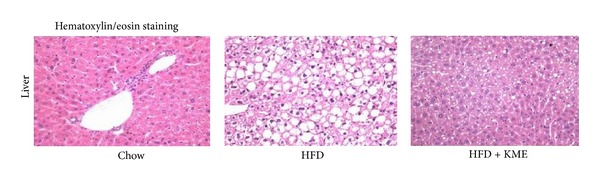
Histologic evaluation of hepatic steatosis. Hematoxylin and eosin staining of liver from mice fed chow diet (c), high-fat diet (HFD), or high-fat diet supplemented with KME at 3000 mpk (HFD + KME) (original magnification 400x). High-fat diet mice had severe hepatic steatosis. High-fat diet-induced mice fed KME at 3000 mpk had significantly reduced evidence of hepatic steatosis.

One of the most common characteristics among people with obesity is the development of fatty liver [33, 34]. Therefore, we also analyzed the effect of KME on fatty liver development. Histologic evaluation is regarded as the “gold standard” for assessing the presence and severity of NAFLD [35]. We histologically evaluated liver sections to determine the extent to which KME attenuated hepatic steatosis development. As shown in Figure 4, the control group exhibited little histologic evidence of hepatic steatosis. Severe steatosis was observed in mice fed a high-fat diet without KME. However, a marked reduction in the degree of steatosis was seen in livers from high-fat diet mice treated with KME. Furthermore, hepatic steatosis scores were dramatically lower in the high-fat diet mice fed KME (grade 1).

### 3.5. Effect of KME on mRNA Levels of Adipogenic Factors in 3T3-L1 Adipocytes

**Figure 5:**
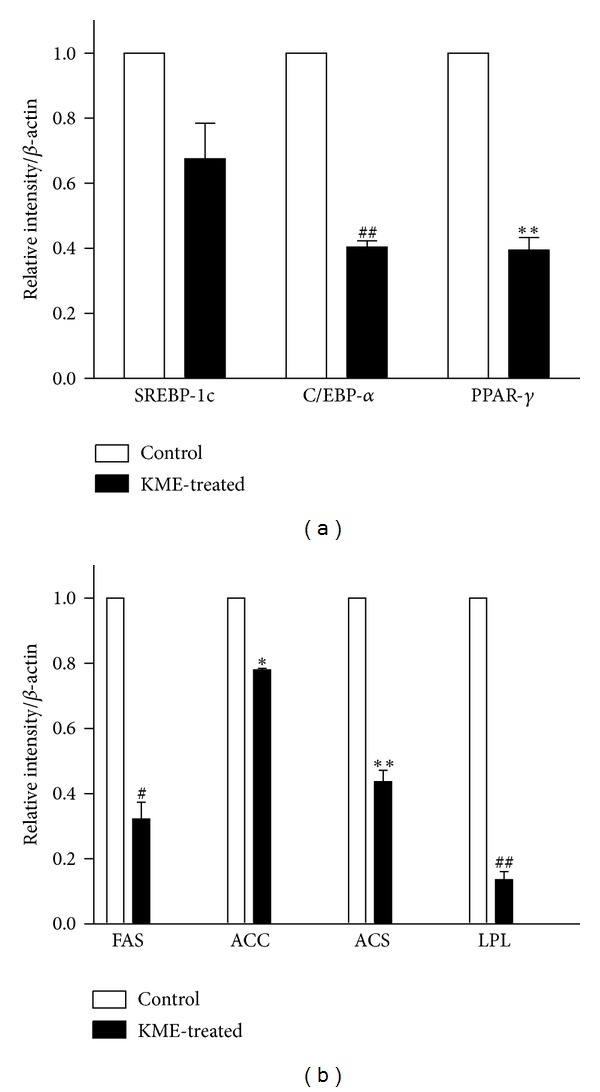
mRNA expressions of adipogenic enzymes and transcription factors in KME-treated 3T3-L1 adipocytes. On day 8 after differentiation, adipocytes were cultured in 6-well plates with or without KME (6 *μ*g/*μ*L) for 18 hours. Graphs represent mRNA expression of (a) adipogenic enzymes ACC, ACS, FAS, and LPL and (b) adipogenic transcription factors SREBP-1c, C/EBP-*α*, and PPAR-*γ*. Data are expressed relative to untreated control cells and represent the mean ± SEM (*P < 0.05, ^#^ *P* < 0.01, **P < 0.005, ^##^ *P* < 0.001, *n* = 3).

To demonstrate the inhibitory mechanism of KME on adipocyte differentiation, we measured the expression levels of the transcription factors PPAR-*γ*, C/EBP-*α*, and SREBP-1c in 3T3-L1 cells treated with 6 *μ*g/*μ*L of KME. Our results show that PPAR-*γ*, C/EBP-*α*, and SREBP-1c mRNA levels were decreased by 64%, 60%, and 32%, respectively, (Figure 5(a)). We also measured the mRNA expression levels of adipogenic enzymes, such as FAS, ACS, and ACC. We found that they were reduced by 69%, 55%, and 22%, respectively (Figure 5(b)). LPL expression is not directly associated with lipid synthesis but is strongly associated with adipogenesis. Therefore, we measured the expression level of LPL mRNA and found that the level of expression in KME-treated 3T3-L1 cells was decreased by 89% compared to that of untreated cells (Figure 5(a)).

## 4. Discussion

Our study is the first to demonstrate that KME prevents weight gain in mice. We examined the effects of KME on HFD-induced obesity in C57Bl/6 mice. Our results showed that body weight gain in groups fed a diet supplemented with KME was reduced compared to control HFD mice. This effect was more distinct in animals fed a high-fat diet. Epididymal WAT in C57Bl/6 mice was significantly reduced by KME supplementation. This study also provides evidence that dietary supplementation of KME protects against hepatic steatosis development. We have considered the possibility that the effect of KME may be mediated by food intake because decreased food intake would be expected to significantly affect body weight, which influences hepatic steatosis. In this study, however, food intake did not differ between the groups. This suggests that KME directly protected against obesity and hepatic steatosis independent of food intake. 

Brown adipose tissues (BATs) use stored triacylglycerols (TG) to maintain body temperature. These cells produce energy from fatty acid metabolism to generate heat through the action of uncoupling protein 1 (UCP1), a mitochondrial protein found only in brown adipose tissue. Brown adipocytes contain less TG and more mitochondria than white adipocytes, resulting in their unique color. KME treatment increased UCP1 expression levels in BAT, which poised the mitochondria for respiration uncoupling [36]. This effect is consistent with the observed enhancement of cold tolerance and helps explain the increase in energy expenditure and resistance to weight gain. 

Mitochondrial function can affect total body metabolism. This is most evident in muscle, a metabolically flexible tissue that switches between lipid and carbohydrate substrates to fulfill energy requirements [37]. One study found that mitochondrial OXPHOS activity within the oxidative *soleus *muscle, which is resistant to fatigue and is dependent on mitochondrial activity for ATP production, was more affected than in the glycolytic muscle *tibialis anterior*. The main changes included a reduction in respiratory chain activity with a concomitant decrease in mitochondrial ATP production [38]. In line with this finding and previously [15], KME significantly improved muscle oxidative capacity, resulting in enhancing the endurance.

To further consolidate our hypothesis that KME inhibits obesity, we evaluated the effects of KME treatment *in vitro* using an adipocyte differentiation development system, which is regulated by transcriptional activators such as PPAR-*γ*, C/EBP-*α*, and some transcriptional regulators at the molecular level [39, 40]. PPAR-*γ* is a member of the nuclear receptor superfamily of transcription factors and is dominantly expressed in adipose tissue. These transcription factors appear to be the main activators of adipocyte differentiation [41].

The C/EBPs belong to a large family of leucine zipper transcription factors [42]. C/EBP-*α* is a promising candidate transcription factor for directly controlling adipocyte differentiation [43].

SREBP1 is known to critically cross-activate a ligand binding domain of PPAR-*γ* and activate the production of an endogenous PPAR-*γ* ligand [27]. SREBP1 also regulates the expression of enzymes involved in fatty acid desaturation and lipogenesis.

Adipocyte differentiation involves a series of programmed changes in the expression of specific genes. Adipogenesis can be induced through the action of some enzymes, such as FAS, ACC, and acyl-CoA synthetase (ACS). The expressions of these genes are regulated by transcription factors, including PPAR-*γ*, C/EBP-*α*, and SREBP1, which are known to be crucial activators for adipogenesis and showed early changes in gene expression during adipocyte differentiation [44, 45]. In this study, we showed that KME treatment significantly decreased SREBP-1c, C/EBP-*α*, and PPAR-*γ* mRNA expression in cultured 3T3-L1 adipocytes and inhibited expression of adipocyte-specific proteins (FAS, ACC, ACS, and LPL). Collectively, these observations suggest that KME suppresses adipocyte differentiation by regulating the expression levels of adipogenic transcription factors.

Further investigation is necessary to define specific mechanisms by which KME protects against obesity-mediated hepatic steatosis. To elucidate the mechanism associated with the antiobesity effects of KME, additional studies should be conducted with the most abundant component in KME. Sung [46] reported that pentacyclic triterpenoid oleanolic acid has antiobesity activity. We have shown that KME contains oleanolic acid, which has anticancer activity [47]. It is possible that oleanolic acid might be the leading antiobesity component in KME. 

## 5. Conclusion

KME had marked inhibitory effects on the development of obesity and NAFLD in mice fed high-fat diets. Activating endurance capacity and increasing thermogenesis are two possible mechanisms for the antiobesity effect of KME. This finding suggests that KME may be a potential dietary strategy for preventing obesity and NAFLD.

## Figures and Tables

**Table 1:** 

Gene name	Accession number		Sequence
ACC	AY451393	Forward	5′-GAG TGA CTG CCG AAA CAT CTC TG-3′
		Reverse	5′-GCC TCT TCC TGA CAA ACG AGT-3′
ACS	NM_007981	Forward	5′-TGA CCT CTC CAT GCA GTC AG-3′
		Reverse	5′-GAG CCT ATG CAC TCA GCC AGT-3′
FAS	NM_007988	Forward	5′-TGG GTT CTA GCC AGC AGA GT-3′
		Reverse	5′-TAC CAC CAG AGA CCG TTA TGC-3′
LPL	BC003305	Forward	5′-GCC CAG CAA CAT TAT CCA GT-3′
		Reverse	5′-GGTCAG ACT TCC TGC TAC GC-3′
SREBP-1c	8C056922	Forward	5′-AAT GGT CCA GGC AAG TTC TGG GT-3′
		Reverse	5′-TCC CTC TCA GCT GTG GTG GTG AA-3′
C/EBP-*α*	M62362	Forward	5′-TGC TGG AGT TGA CCA GTG AC-3′
		Reverse	5′-AAA CCA TCC TCT GGG TCT CC-3′
PPAR-*γ*	NM_011146	Forward	5′-TTT TCA AGG GTG CCA GTT TCA ATC C-3′
		Reverse	5′-AAT CCT TGG CCC TCT GAG AT-3′
UCP1	NM_009463	Forward	5′-GGC CCT TGT AAA CAA CAA AAT AC-3′
		Reverse	5′-GGC AAC AAG AGC TGA CAG TAA AT-3′
*β*-Actin	NM_007393	Forward	5′-GAC TAC CTC ATG AAG ATC-3′
		Reverse	5′-GAT CCA CAT CTG CTG GAA-3′

ACD: acyl-coenzyme A dehydrogenase; ACO: acyl-coenzyme A oxidase; ACS: acetyl-coenzyme A synthetase; COX-2: cyclooxygenase 2; CPT-1: carnitine palmitoyltransferase 1; FAS: fatty acid synthase; HSL: hormone-sensitive lipase; IL-6: interleukin-6; LPL: lipoprotein lipase; MCP1: monocyte chemotactic protein 1; SREBP-1c: sterol regulatory element binding protein-1.
